# Effects of comprehensive nursing intervention on quality of life, Self-efficacy, gastrointestinal reaction and immune function of patients with breast cancer undergoing chemotherapy

**DOI:** 10.12669/pjms.40.6.7857

**Published:** 2024-07

**Authors:** Fang Hao, Cui-xin Guo, Ya-min Zhang, Shu-yun Guo

**Affiliations:** 1 Fang Hao, Department of General Surgery, The Second Hospital of Hebei Medical University, Shijiazhuang 050000, Hebei, China; 2 Cui-xin Guo, Department of Ophthalmology, Shijiazhuang People’s Hospital, Shijiazhuang 050000, Hebei, China; 3 Ya-min Zhang, Dept. of Pediatrics, 980 Hospital of Joint Service Support Force, Shijiazhuang 050000, Hebei, China; 4 Shu-yun Guo, Department of Vascular Surgery, The Second Hospital of Hebei Medical University, Shijiazhuang 050000, Hebei, China

**Keywords:** Comprehensive nursing intervention, Breast cancer, Chemotherapy, Quality of life, Immune function

## Abstract

**Objective:**

To assess the effects of comprehensive nursing intervention on quality of life, self-efficacy, gastrointestinal reaction and immune function of patients with breast cancer undergoing chemotherapy.

**Methods:**

This was a retrospective study. One hundred and twenty patients receiving chemotherapy after breast cancer surgery were randomly divided into the experimental group and the control group(n=60) from January 2021 to January 2023. Patients in the perioperative period, the experimental group were given comprehensive nursing intervention, while those in the control group were given conventional specialist nursing intervention. The differences in quality of life, self-efficacy, gastrointestinal reaction, immune function and patient satisfaction between the two groups were compared and analyzed.

**Results:**

After the intervention, the SF-36 scores in the experimental group were significantly higher than those in the control group (P=0.00), the efficacy indicators were significantly improved compared to the control group(P=0.00); the scores of gastrointestinal symptoms in the experimental group were significantly lower than those in the control group after the intervention(P<0.05). The indexes of CD3^+^, CD4^+^ and CD4^+^/CD8^+^ in the experimental group after the intervention were significantly higher than those in the control group(P=0.00); The patient satisfaction in the experimental group was 100%, which was significantly higher than 92% in the control group, with statistically significant differences(P=0.02).

**Conclusion:**

Comprehensive nursing intervention leads to a variety of benefits in the treatment of patients with breast cancer during postoperative chemotherapy, such as relieving patients’ gastrointestinal reactions, improving their immune function and quality of life, besides effectively improving their self-efficacy, which is worthy of clinical application.

## INTRODUCTION

Breast cancer is a malignant tumor that occurs in the epithelial tissue of the breast gland and is highly prevalent in women, with an incidence rate of 7%-10%.^1^ The change of people’s diet and lifestyle has contributed to the increasing incidence of breast cancer, which is a serious threat to women’s life and health. Given its complex causes, such as genetics, lifestyle, marital status, and obesity,^2^ there are many barriers to a clinical cure for breast cancer.^3^

Breast cancer is clinically manifested as breast mass, nipple collapse, nipple discharge, usually bloody discharge, and skin changes, and in advanced stages, axillary lymph node metastases and distant metastases to lung, bone, brain and liver, posing a serious threat to the life safety of patients.^4^ Currently, surgery and chemotherapy are clinically preferred for the treatment of breast cancer.^5^ Chemotherapy, as the main means to prevent tumor metastasis and recurrence after surgery, leads to effectively killing cancer cells and achieving the purpose of relieving patients’ clinical symptoms.^6^ However, it has been proved in practice that while killing cancer cells in patients, it will also kill normal cells, resulting in reduced immune function and gastrointestinal adverse effects such as nausea and vomiting.

In this case, patients’ quality of life and clinical outcomes will be remarkably reduced. In the past, specialized nursing care has placed too much emphasis on the recovery of the surgical incision, resulting in a slow recovery of the patient. With the rapid development of nursing science, comprehensive nursing model, an intervention method based on a continuum of thinking with many advantages, such as personalization, holistic and effective, is also widely used in clinical practice.^7^ In this study, comprehensive nursing intervention measures were implemented for patients with breast cancer during postoperative chemotherapy, to assess the effects of comprehensive nursing intervention on quality of life, self-efficacy, gastrointestinal reaction and immune function of patients with breast cancer undergoing chemotherapy.

## METHODS

This was a retrospective study. One hundred and twenty patients receiving chemotherapy after breast cancer surgery were randomly divided into the experimental group and the control group, with 60 cases in each group from January 2021 to January 2023. Patients in the experimental group were given comprehensive nursing intervention in the perioperative period, while those in the control group were given conventional specialist nursing intervention in the same period. No statistically significant differences were observed on the comparison of general data between the two groups, which were comparable (Table-I).

**Table-I:** Comparative analysis of general data of the experimental group and the control group (*χ̅*±*S*) n=60.

Index	Experimental group	Control group	t/χ^2^	P
Age (years)	65.43±4.45	66.25±3.62	1.10	0.27
Medical history (months)	19.25±3.51	18.68±3.78	0.85	0.40
Pathological stage				
Stage I (cases, %)	15 (%)	13 (%)	0.19	0.67
Stage II (cases, %)	40 (%)	39 (%)	0.04	0.85
Stage III (cases, %)	5 (%)	8 (%)	0.78	0.38
Surgical method			0.32	0.57
Radical surgery (cases, %)	52 (%)	54 (%)		
Breast conserving surgery (cases, %)	8 (%)	6 (%)		
Marital status			0.37	0.54
Married (cases, %)	53 (%)	55 (%)		
Unmarried (cases, %)	7 (%)	5 (%)		

P>0.05.

### Ethical Approval:

The study was approved by the Institutional Ethics Committee of The Second Hospital of Hebei Medical University on June 29, 2021(No.: 2022-R113), and written informed consent was obtained from all participants.

### Inclusion criteria:

- Patients diagnosed with breast cancer after postoperative pathological examination and requiring further chemotherapy,^8^
- Patients with stable disease without serious complications or other organic diseases,
- Patients with clear consciousness, no mental disorders, and able to actively cooperate with the implementation of treatment and nursing programs,
- Patients aged 55~75 years,
- Patients who were informed of the experimental content and voluntarily signed the informed consent form,
- Patients with complete clinical data,
- Patients who were able to cooperate with the completion of the study and had good treatment compliance.

### Exclusion criteria:

- Patients with poor treatment compliance and unable to cooperate with treatment or nursing,
- Patients with severe cognitive dysfunction or a history of mental illness,
- Patients with other vital organs such as heart, liver and kidney dysfunction,
- Patients with cancer metastasis and survival time <one year.

Patients in the control group were given a routine care plan, including a general physical examination after admission and a chemotherapy plan based on the examination results. Patients and their families were given a manual on breast cancer chemotherapy to improve their knowledge of breast cancer and chemotherapy. During chemotherapy, patients’ vital signs were closely monitored, and adverse reactions were reported to the doctor and nursing interventions were given accordingly. In addition, communication between nursing staff and patients was strengthened to understand patients’ psychological status and provide targeted psychological care.

Patients in the experimental group were given a comprehensive nursing intervention model, including:

### Zsychological care:

The nursing staff took the initiative to communicate with the patients and implemented personalized psychological counseling according to the psychological characteristics of female patients to relieve their psychological pressure and eliminate their bad emotions. At the same time, the patients’ psychological activities were closely observed, their family members were instructed to encourage and comfort them, and communication between patients was strengthened through regular patient communication meetings or the establishment of WeChat groups, to improve the degree of cooperation in treatment, Moreover, the patients were instructed to wear wigs and breast prostheses to avoid the negative emotions caused by hair loss and breast loss due to chemotherapy.

### Health education:

Patients were promptly given a health education booklet on breast cancer after admission, and were taught about surgery and chemotherapy. In addition, they were also introduced to the prevention and treatment of adverse reactions such as nausea, vomiting and hair loss during chemotherapy.

### Dietary guidance:

Personalized healthy diet recipes were developed by nursing staff according to patients’ individual dietary preferences and habits to encourage them to follow relevant dietary principles: supplementation of protein-rich foods such as lean meat, eggs and fish, supplementation of anti-cancer foods such as seaweed, shiitake mushrooms and fungus, and abstinence from spicy and other stimulating foods. At the same time, patients were advised to eat a small amount of easily digestible food before and after chemotherapy.

### Exercise:

Patients are encouraged to take appropriate exercise such as walking, race walking, jogging and rehabilitation exercises to improve their immunity, promote their blood circulation, enhance gastrointestinal peristalsis, reduce diarrhea, constipation and other digestive tract discomfort symptoms, In addition, the patients were supervised during exercise and did the amount of exercise they could tolerate.

### Observation indexes:

### Comparative analysis of quality of life after intervention:

The 36-item Short-Form (SF-36)^9^ was utilized to evaluate and compare the quality of life of the two groups, which mainly included five dimensions of emotional function, role function, cognitive function, physical function and social function. The higher the score, the better the quality of life,^10^

### Comparative analysis of self-efficacy:

The Strategies Used by People to Promote Health (SUPPH) was employed to evaluate the two groups of patients before and after the intervention. The reliability, content validity and structure validity of the scale were 0.928, 0.875 and 0.723 respectively. Patients’ self-efficacy was evaluated from three dimensions: positive attitude (15 items), stress reduction (10 items), and decision making (3 items), with a score of 1-5 for each item. The score was positively correlated with self-efficacy.

### Comparison of gastrointestinal reactions:

The gastrointestinal reactions (appetite, eating, nausea and vomiting, constipation) of the two groups during chemotherapy were evaluated according to the Classification Standard for Common Toxic and Side Effects of Anticancer Drugs (WHO). The standard uses a 5-level score ranging from 0 to 4, with higher scores indicating more severe symptoms.^11^

### Comparative analysis of immune function:

Venous blood was extracted from patients before and after the intervention, and blood samples were collected in all cases under fasting condition in the morning to detect T lymphocyte subsets CD3^+^, CD4^+^, CD8^+^ and CD4^+^/CD8^+^ by flow cytometry, and the changes of the above indexes were compared and analyzed.

### The Short-Form Patient Satisfaction Questionnaire

(PSQ-18)^12^ was used to compare and analyze the patients’ satisfaction before and after the intervention, including very satisfied, relatively satisfied, satisfied, uncertain and dissatisfied. Total satisfaction =(very satisfied + relatively satisfied + satisfied)/total number of cases x 100%.The follow-up work of all patients was completed by the same group of surgeons.

### Statistical analysis:

All data in this study were statistically analyzed by SPSS 20.0 software, and measurement data were expressed as(*χ̅*±*S*), the confidence interval was 95%. Two independent sample t test was used for comparison between groups, paired t test was used to analyze data within groups, and χ^2^ test was used for the comparison of rates. P<0.05 indicates a statistically significant difference.

## RESULTS

The scores of emotional functions, role function, cognitive function, somatic function and social function of the experimental group after intervention were significantly higher than those of the control group, with statistically significant differences(p=0.00) (Table-II).

**Table-II:** Comparative analysis of the quality of life scores between the two groups after the intervention (*χ̅*±*S*) n=60.

Group	Emotional function	Cognitive function	Somatic function	Role function	Social function
Experimental group	7.52±1.02	5.42±1.06	8.75±1.13	5.42±0.62	4.48±0.62
Control group	6.10±1.04	4.37±1.06	6.43±1.092	4.74±0.52	3.60±0.49
t	7.56	5.43	11.41	6.57	8.60
p	0.00	0.00	0.00	0.00	0.00

*P<0.05.

No statistically significant differences were observed between the two groups in positive attitude, stress reduction, decision making and other indicators before the intervention(P>0.05). After the intervention, the above indexes of the experimental group were significantly improved compared with the control group, with a statistically significant difference(P=0.00) (Table-III).

**Table-III:** Comparative analysis of emotional status of the two groups before and after the intervention (*χ̅*±*S*) n=60.

Index		Experimental group*	Control group	t	p
Positive attitude	Before intervention	42.68±5.06	43.03±5.35	0.37	0.72
	After intervention*	52.75±5.53	45.23±5.13	7.71	0.00
Stress reduction	Before intervention	24.07±8.07	23.75±7.33	0.23	0.82
	After intervention*	38.87±7.21	27.50±6.90	8.83	0.00
Decision making	Before intervention	7.95±2.55	7.83±1.88	0.29	0.78
	After intervention*	12.88±1.72	9.03±1.90	11.66	0.00

* P<0.05.

No statistically significant differences were observed between the two groups in the scores of gastrointestinal reactions (appetite, eating, nausea and vomiting, constipation) (P>0.05). After the intervention, the scores of gastrointestinal symptoms in the experimental group were significantly lower than those in the control group, with a statistically significant difference(P<0.05) (Table-IV).

**Table-IV:** Comparative analysis of gastrointestinal reactions between the two groups (*χ̅*±*S*) n=60.

Index		Experimental group*	Control group	t	p
Appetite	Before intervention	3.07±0.36	2.98±0.39	1.21	0.23
	After intervention*	1.38±0.49	1.65±0.48	3.01	0.00
Eating	Before intervention	3.27±0.52	3.20±0.55	0.69	0.49
	After intervention*	0.78±0.42	1.58±0.53	9.20	0.00
Nausea and vomiting	Before intervention	3.02±0.22	2.95±0.34	1.27	0.21
	After intervention*	1.12±0.32	1.28±0.45	2.31	0.02
Constipation	Before intervention	2.37±0.66	2.28±0.67	0.69	0.49
	After intervention*	0.97±0.26	1.08±0.28	2.38	0.02

* P<0.05.

No statistically significant differences were observed in the levels of CD3^+^, CD4^+^, CD8^+^ and CD4^+^/CD8^+^ between the two groups before the intervention (P>0.05). After treatment, CD3^+^, CD4^+^, and CD4^+^/CD8^+^ and other indicators in the experimental group were significantly higher than those in the control group, with a statistically significant difference(P=0.00). There was no significant change in CD8+ in both groups before and after treatment (P>0.05) (Table-V). The patient satisfaction in the experimental group was 100%, which was significantly higher than 92% in the control group, with a statistically significant difference (P=0.02) (Table-VI).

**Table-V:** Comparative analysis of T lymphocyte subsets between the two groups before treatment (*χ̅*±*S*) n=60.

Index		Experimental group	Control group	t	p
CD3+ (%)	Before treatment	41.36±6.88	41.87±5.95	0.43	0.67
	After treatment*	48.97±7.43	44.79±6.88	3.19	0.00
CD4+ (%)	Before treatment	22.37±4.29	22.16±4.12	0.28	0.78
	After treatment*	33.89±4.96	27.74±4.77	6.92	0.00
CD8+ (%)	Before treatment	23.46±3.84	23.75±4.06	0.40	0.70
	After treatment	24.58±3.88	24.27±4.18	0.42	0.68
CD4+/CD8+	Before treatment	0.95±0.08	0.93±0.03	1.96	0.05
	After treatment*	1.39±0.13	1.15±0.10	11.58	0.00

* P<0.05.

**Table-VI:** Comparative analysis of patient satisfaction between the two groups (*χ̅*±*S*) n=60.

Group	Very satisfied	Relatively satisfied	Satisfied	Uncertain	Dissatisfied	Total satisfaction*
Experimental group	42	10	8	0	0	60 (100%)
Control group	30	19	6	0	5	55 (92%)
χ^2^						5.22
P						0.02

* P<0.05.

## DISCUSSION

It was confirmed in our study that there was no significant difference in the levels of positive attitude, stress reduction, decision-making and other self-efficacy indicators between the two groups before intervention(P>0.05). After the intervention, the above indicators in the research group were significantly improved compared with the control group, with statistical significance (P=0.00). This can be attributed to the fact that the comprehensive care intervention improved patients’ knowledge about postoperative breast cancer and their psychological defenses, resulting in higher psychological tolerance, increased self-care awareness and self-postoperative psychological resilience for possible adverse effects and clinical symptoms in patients receiving chemotherapy, which is more conducive to the improvement of patients’ self-efficacy.

Previous specialized nursing tends to carry out simple life health guidance and oral education for patients, but does not give consideration to patients’ physical and mental health and other comprehensive conditions. Therefore, it cannot be personalized and systematic, and has little impact on patient compliance with poor effect. Comprehensive nursing intervention, a new nursing method, provides nursing care according to the predetermined method for a certain disease combined with the specific situation of the patient.^13^ It is more operational and easily accepted by patients than traditional nursing, and is a more practical and perfect model of nursing.^14^ It attaches more importance to the psychological care and comfort of patients, and is more personalized and humanized.^15^

Self-efficacy is the embodiment of patients’ self-care ability and self-health awareness in the process of clinical treatment.^16^ It has a close bearing on medical compliance and mental resilience in the course of clinical treatment. For patients with breast cancer, compliance behavior during post-operative chemotherapy helps them to administer medication in a timely manner, regulate their bodies, and respond to physical abnormalities in a stressful manner, which is of significant clinical significance in improving patients’ treatment efficiency and reducing the risk of cancer recurrence.^17^ Comprehensive nursing intervention is a patient-centered nursing intervention that pays attention to the objective needs, subjective wishes and habits of patients and provides them with targeted and comprehensive nursing services. It’s a modern nursing model that fits in with the modern nursing philosophy.^18^ It has been found in studies^19^ that the psychological activities of patients will act on the endocrine system through the central nervous system, thereby affecting the immune function and physiological activities of the body. Based on this, psychological intervention for patients can effectively eliminate their anxiety, depression and other adverse psychology, improve the quality of life of patients. In addition, it can significantly improve patients’ medical experience, increase their satisfaction and cognition of the disease, reduce negative emotions, and increase their trust in and satisfaction with medical staff. It was shown in our study that after the implementation of psychological nursing intervention in the comprehensive group, the SF-36 score index of patients was significantly higher than that of the control group(P=0.00). The patient satisfaction in the experimental group was 100%, which was significantly higher than 92% in the control group, with a statistically significant difference (P=0.02). In addition, the patients were given timely breast cancer health education manuals and informed about the disease, surgery and chemotherapy related knowledge and precautions. At the same time, female patients were given targeted psychological counseling based on individual psychological characteristics to relieve their psychological pressure. The families of the patients were instructed to offer encouragement and comfort to the patients, so that they could actively face problems such as mastectomy and hair loss after chemotherapy and learn how to self-regulate and deal with them. As shown in the results of this study, the scores of digestive tract symptoms in the experimental group after intervention were significantly lower than those in the control group (P=0.00), suggesting that effective nursing intervention can alleviate the discomfort symptoms of patients. Furthermore, patients were monitored for changes in vital signs and pain, reminded to choose the best time for chemotherapy, and to follow dietary guidelines: supplementing protein-rich and anti-tumor foods. They were also advised to actively engage in moderate aerobic exercise and learn self-care by drinking plenty of water and getting plenty of rest. It was shown in this study that after the intervention, CD3^+^, CD4^+^ and CD4^+^/CD8^+^ indexes were significantly higher in the experimental group than in the control group (P=0.00), indicating the importance of comprehensive nursing intervention for regulating the immune function of patients and reducing gastrointestinal reactions during chemotherapy. Chandratre et al.^19^ concluded that a comprehensive nursing intervention model is beneficial to the harmonious relationship between medical staff and patients, the improvement of patients’ psychological satisfaction and sense of security, and the enhancement of confidence in overcoming the disease. In this way, patients can actively cooperate with medical staff to receive treatment, thus facilitating recovery.

### Limitations:

It includes small sample size and short follow-up time. To address this, more patients will be included in future clinical studies with extended follow-up time, so as to evaluate the advantages and disadvantages of this intervention program more objectively and benefit more patients.

## CONCLUSION

Comprehensive nursing intervention is of great clinical significance in the course of postoperative chemotherapy for breast cancer. It is an effective nursing intervention mode worth popularizing and applying, leads to a variety of benefits, such as relieving patients’ gastrointestinal reactions, ameliorating their psychological status, improving their immune function and quality of life, and effectively enhancing their self-efficacy.

### Authors’ Contributions:

**FH and SG** carried out the studies, participated in collecting data, drafted the manuscript, are responsible and accountab le for the accuracy or integrity of the work.

**CG** performed the statistical analysis and participated in its design.

**YZ** participated in acquisition, analysis, or interpretation of data and drafting the manuscript.

All authors read and approved the final manuscript.

## References

[1] Katsura C, Ogunmwonyi I, Kankam HK, Saha S (2022). Breast cancer:presentation, investigation and management. Br J Hosp Med (Lond).

[2] Kashyap D, Pal D, Sharma R, Garg VK, Goel N, Koundal D (2022). Global Increase in Breast Cancer Incidence:Risk Factors and Preventive Measures. Biomed Res Int.

[3] Chen Z, Sang MX, Geng CZ, Hao W, Jia HQ (2022). Clinical curative effect of neoadjuvant chemotherapy combined with immunotherapy and its impact on immunological function and the expression of ER, PR, HER-2 &SATB1 in HER-2-Positive breast cancer patients. Pak J Med Sci.

[4] Liang Y, Zhang H, Song X, Yang Q (2020). Metastatic heterogeneity of breast cancer:Molecular mechanism and potential therapeutic targets. Semin Cancer Biol.

[5] Xu Y, Chao L, Wang J, Sun Y, Li C (2023). Effect of different chemotherapy schemes on early-stage breast cancer patients with Low HER-2 expression. Pak J Med Sci.

[6] Zhao H, Zhang J, Lu Y, Jin J (2019). Neoadjuvant chemotherapy in combination with surgery in the treatment of local advanced breast cancer. Pak J Med Sci.

[7] Coppell KJ, Abel SL, Freer T, Gray A, Sharp K, Norton JK (2017). The effectiveness of a primary care nursing-led dietary intervention for prediabetes:a mixed methods pilot study. BMC Fam Pract.

[8] Shien T, Iwata H (2020). Adjuvant and neoadjuvant therapy for breast cancer. Jpn J Clin Oncol.

[9] Ware JE, Sherbourne CD (1992). The MOS 36-item short-form health survey (SF-36). I. Conceptual framework and item selection. Med Care.

[10] Lončar I, van Dijk SPJ, van Velsen EFS, Buijk SE, Niemeijer ND, Veeken CJ (2022). Radiofrequency Ablation for Benign Symptomatic Thyroid Nodules in the Netherlands:Successful Introduction of a Minimally Invasive Treatment Option Improving Quality of Life. J Vasc Interv Radiol.

[11] Thakur R, Bajaj JK, Dutta A, Sidhu S (2022). Retrospective Observational Study to Evaluate Causality, Preventability and Severity of Adverse Drug Reaction Associated with Anticancer Drugs in a Tertiary Care Hospital in Northern India. Curr Drug Saf.

[12] Hwang SY, Park S, Kwon Y (2019). Recent therapeutic trends and promising targets in triple negative breast cancer. Pharmacol Ther.

[13] Eastwood J, Maitland-Scott I (2020). Patient Privacy and Integrated Care:The Multidisciplinary Health Care Team. Int J Integr Care.

[14] Bardin T, Chalès G, Pascart T, Flipo RM, Korng Ea H, Roujeau JC (2016). Risk of cutaneous adverse events with febuxostat treatment in patients with skin reaction to allopurinol. A retrospective, hospital-based study of 101 patients with consecutive allopurinol and febuxostat treatment. Joint Bone Spine.

[15] Kaul S, Gupta M, Bandyopadhyay D, Hajra A, Deedwania P, Roddy E (2021). Gout Pharmacotherapy in Cardiovascular Diseases:A Review of Utility and Outcomes. Am J Cardiovasc Drugs.

[16] Klassen RM, Klassen JRL (2018). Self-efficacy beliefs of medical students:a critical review. Perspect Med Educ.

[17] Awick EA, Phillips SM, Lloyd GR, McAuley E (2017). Physical activity, self-efficacy and self-esteem in breast cancer survivors:a panel model. Psychooncology.

[18] Coppell KJ, Abel SL, Freer T, Gray A, Sharp K, Norton JK (2017). The effectiveness of a primary care nursing-led dietary intervention for prediabetes:a mixed methods pilot study. BMC Fam Pract.

[19] Chandratre P, Mallen C, Richardson J, Muller S, Hider S, Rome K (2018). Health-related quality of life in gout in primary care:Baseline findings from a cohort study [published correction appears in Semin Arthritis Rheum 2022; 52 151800]. Semin Arthritis Rheum.

